# The emergent role of mitochondrial surveillance in cellular health

**DOI:** 10.1111/acel.13710

**Published:** 2022-09-11

**Authors:** Elissa Tjahjono, Daniel R. Kirienko, Natalia V. Kirienko

**Affiliations:** ^1^ Department of BioSciences Rice University Houston Texas USA

**Keywords:** aging, mitochondria, mitochondrial membrane transport proteins, mitophagy, physiological stress, reactive oxygen species, surveillance

## Abstract

Mitochondrial dysfunction is one of the primary causatives for many pathologies, including neurodegenerative diseases, cancer, metabolic disorders, and aging. Decline in mitochondrial functions leads to the loss of proteostasis, accumulation of ROS, and mitochondrial DNA damage, which further exacerbates mitochondrial deterioration in a vicious cycle. Surveillance mechanisms, in which mitochondrial functions are closely monitored for any sign of perturbations, exist to anticipate possible havoc within these multifunctional organelles with primitive origin. Various indicators of unhealthy mitochondria, including halted protein import, dissipated membrane potential, and increased loads of oxidative damage, are on the top of the lists for close monitoring. Recent research also indicates a possibility of reductive stress being monitored as part of a mitochondrial surveillance program. Upon detection of mitochondrial stress, multiple mitochondrial stress‐responsive pathways are activated to promote the transcription of numerous nuclear genes to ameliorate mitochondrial damage and restore compromised cellular functions. Co‐expression occurs through functionalization of transcription factors, allowing their binding to promoter elements to initiate transcription of target genes. This review provides a comprehensive summary of the intricacy of mitochondrial surveillance programs and highlights their roles in our cellular life. Ultimately, a better understanding of these surveillance mechanisms is expected to improve healthspan.

## Graphical abstract

Mitochondrial dysfunction is a primary contributor to many pathologies, including neurodegenerative diseases, cancer, metabolic disorders, and aging. Various indicators of unhealthy mitochondria, including halted protein import, dissipated membrane potential, and increased loads of oxidative damage, are closely monitored by surveillance mechanisms to anticipate possible havoc within these multifunctional organelles. This review provides a comprehensive summary of the intricacy of mitochondrial surveillance programs and highlights their roles in our cellular life.

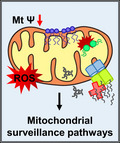

## 1 INTRODUCTION

The field of mitochondrial surveillance has burgeoned within the last 20 years as recognition of the contribution of mitochondrial dysfunction to chronic health issues has increased. In retrospect, it seems apparent that at least three factors would drive intense cellular scrutiny of mitochondria. First, they are responsible for the generation of much of the ATP in the cell, along with β‐oxidation of fatty acids, lipid metabolism, and amino acid catabolism (Spinelli & Haigis, 2018). They are also a central regulator of apoptosis. Disruption of any of these events is likely to be lethal for the cell. Second, mitochondrial biochemistry produces reactive oxygen species (ROS) (Nissanka & Moraes, 2017) and other toxic metabolic intermediates like methylmalonate or propionate (Fernandez‐Gomez et al., 2005). Finally, mitochondria originated as free‐living bacteria that became engulfed within other cells (Gray, 2012). Although these uneasy bedfellows eventually navigated their way toward a symbiotic relationship, it likely required them to steer a course through a transition period of close contact despite potential danger.

Due to these three factors, mitochondria and cells underwent massive changes. First, most of the genes encoding the mitochondrial proteome migrated from this organelle to the nuclear genome (Anderson et al., 1981; Li et al., 2009; Taanman, 1999). This reduction of mitochondrial genome content was a major adaptation event during the transition from an independent bacterium into an endosymbiotic organelle. The smaller genome size provides replication or survival advantage for the organelle and gives the host additional flexibility in regulating their expression. However, this gene transfer limits the organelles' capability to live outside their hosts and the deleterious effect of mutant mitochondrial DNA (mtDNA) propagation.

Second, mitochondrial signaling functions to recognize damage‐associated molecular patterns (mtDAMPs), including molecular motifs from their prokaryotic origin (e.g., N‐formyl peptides and mtDNA) and various mitochondrial metabolites such as cytochrome *c*, ATP, and cardiolipin, among others, released during organellar stress or damage (Grazioli & Pugin, 2018). These molecules induce inflammatory responses and ROS production (Hazeldine et al., 2015; Oka et al., 2012; Raoof et al., 2010).

Finally, cells develop surveillance mechanisms that are the molecular equivalent of the Cold War‐era disarmament policy “*trust, but verify*”. Mitochondria are heavily monitored to limit potential damage and to preserve functions. Mitochondrial surveillance pathways orchestrate expression of tens to hundreds of genes via mitochondria‐to‐nuclear communication, also known as mitochondrial retrograde signaling. Curiously, despite the importance of mitochondrial quality control mechanisms, this biological phenomenon is relatively understudied. Although several pathways were found to respond to mitochondrial damage, only one, the mitochondrial unfolded protein response (UPR^mt^) (Haynes et al., 2013; Haynes & Ron, 2010; Naresh & Haynes, 2019), has been extensively studied, while the others have only recently been published. Many of these pathways monitor compromised mitochondrial protein import. Others monitor membrane potential, redox imbalance, mitochondrial bioenergetics, ceramide, mevalonate, and lipid biosynthesis. The next sections will discuss the regulations of these mitochondrial surveillance programs.

## 2 WHEN IMPORT FAILS: PERTURBATIONS OF PROTEOSTASIS AS A SIGNAL OF MITOCHONDRIAL DISRUPTION

Typical modern mitochondrial genome consists only of a group of rRNAs, tRNAs, and a small handful of proteins involved in the electron transport chain (ETC) (Anderson et al., 1981). This is inconvenient, as ~ 99% of the proteins required for normal mitochondrial function are now encoded in the nuclear genome (Anderson et al., 1981; Li et al., 2009; Taanman, 1999), while other components are still in the mitochondrial genome. ETC complex subunits are required in proper stoichiometric ratios to avoid the assembly of non‐functional complexes, some of which may have dominant‐negative function (i.e., assembly of incomplete complexes can interact with substrates but not carry out function). As such, the cell requires careful coordination between the two genomes to ensure that this does not happen.

**FIGURE 1:**
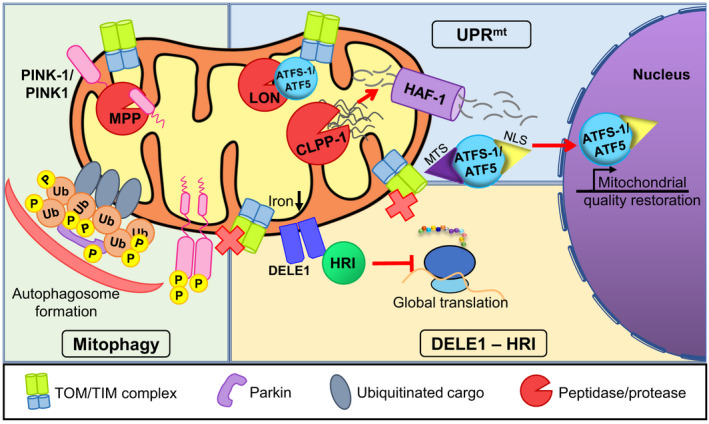
Failed mitochondrial import activates proteostatic surveillance. Several pathways in the cell monitor mitochondrial import and are activated by its failure, including the UPR^mt^, the PINK1/parkin mitophagic axis, and the DELE1‐HRI pathway. Although the details vary (see text), these pathways are generally activated when proteins are not properly imported into mitochondria. Often this serves as a direct signal of mitochondrial damage, stimulating transcriptional changes to restore homeostasis, limit protein translation and import, promote mitochondrial recycling via mitophagy, and even activate cell death pathways. Abbreviations: MTS—mitochondrial targeting sequence, NLS—nuclear localization signal, P—Parkin, and Ub—ubiquitin

**FIGURE 2:**
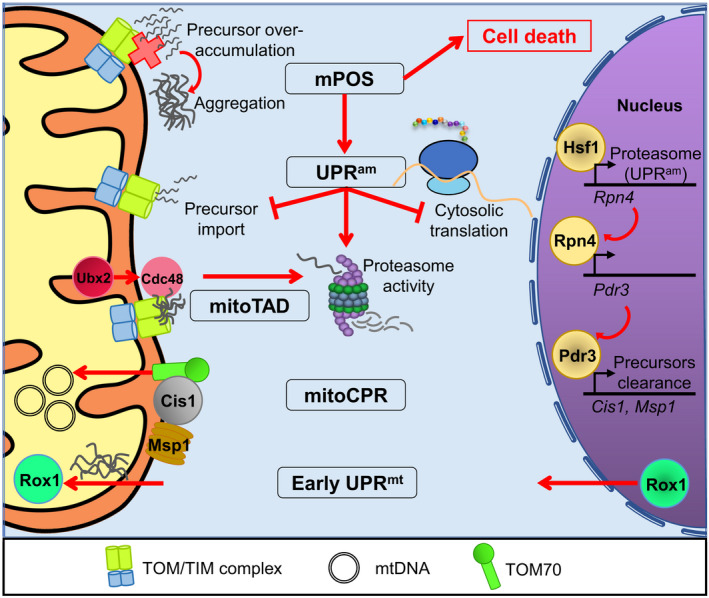
Novel mitochondrial import surveillance pathways identified in yeast. The mitochondrial precursors over‐accumulation stress, indicated by aggregation of mitochondrial precursors or clogged import channel, activates multiple response pathways in yeast. These pathways include the UPR^am^, mitoTAD, mitoCPR, and the mPOS‐mediated cell death pathways. These pathways often act to increase proteasomal activity to prevent protein aggregation and to limit protein import. In addition, an early UPR^mt^ is also activated at the first detection of protein aggregates in the mitochondria to increase cell survival

Relocation of mitochondrial protein genes to the nucleus means that most of the proteins for mitochondrial function must be trafficked to and imported into mitochondria. These barrier‐crossing processes may require unfolding of proteins facilitated by mtHsp70 and refolding upon arrival in the matrix (Avendaño‐Monsalve et al., 2020; Bykov et al., 2020; Sato et al., 2019). Essentially all these materials utilize the well‐understood TOM (translocase of outer membrane) and TIM (translocase of inner membrane) complexes (Wasilewski et al., 2017; Wiedemann & Pfanner, 2017), which leverage the electrochemical proton gradient generated by ETC to facilitate import. This process is complicated and energetically intensive. Cellular stresses could limit import and stalled polypeptides will accumulate in the mitochondrial membrane, disturbing mitochondrial protein homeostasis (proteostasis). For all these reasons, mitochondrial import represents a valuable target used by several different surveillance systems (Figures 1, 2).

Several of these surveillance systems restore proteostasis by reducing protein synthesis. These pathways include the UPR^mt^, the integrated stress response (ISR), and the yeast UPR^am^ (UPR^mt^ activated by mistargeting of proteins). Arguably, the most widely known of these is the UPR^mt^, an evolutionarily conserved response to aberrations in mitochondrial transport. Initially, the UPR^mt^ was discovered due to the introduction of a mutated, improperly folding mitochondrial matrix protein, to specifically induce the mitochondrial chaperones *Cpn60*, *Cpn10*, and several other mitochondrial resident proteins (Martinus et al., 1996; Zhao et al., 2002). Deletion or depletion of mtDNA also had this effect. Promoter analysis identified a CHOP (C/EBP homology protein [TGCAATC])‐binding element in these genes, and a heterodimer of CHOP and C/EBP‐β was shown to be responsible for their regulation (Zhao et al., 2002). Further upstream, mitochondrial stress activates c‐Jun (through JNK signaling) to bind the AP‐1 element found in the promoter of CHOP and C/EBP‐β (Horibe & Hoogenraad, 2007).

This pathway has been more comprehensively researched in *Caenorhabditis elegans*. One of the central actors in the nematode's UPR^mt^ network is the bZIP‐family transcription factor ATFS‐1. Due to the presence of an N‐terminal mitochondrial localization sequence, ATFS‐1 is trafficked to mitochondria where it is imported and rapidly degraded by matrix‐resident proteases (Haynes et al., 2013; Haynes & Ron, 2010; Nargund et al., 2012). This process can be disrupted if mitochondrial chaperones (such as HSP‐6 and HSP‐60) are overwhelmed by excess of unfolded proteins (Yoneda, 2004). In this case, the resident proteases, particularly CLPP‐1, will begin to cleave the misfolded proteins, whose peptide fragments are then exported by HAF‐1, a mitochondrial ABC transporter. It is also speculated that charged peptides exported by HAF‐1 may affect mitochondrial membrane potential (Rolland et al., 2019). Combination of these events compromises import efficiency, leading to the accumulation of ATFS‐1 in the cytoplasm. This allows a secondary, weaker, nuclear localization signal in ATFS‐1 to reroute the transcription factor to the nucleus (Haynes et al., 2010; Haynes & Ron, 2010; Nargund et al., 2012).

Once in the nucleus, ATFS‐1 works with DVE‐1, LIN‐65, MET‐2, and UBL‐5 to drive expression of mitochondrial chaperones and other repair machinery (~500 genes in total) to promote longevity and stress tolerance and restore proteostasis (Benedetti et al., 2006; Haynes et al., 2007; Haynes et al., 2010; Haynes et al., 2013). The UPR^mt^ also modulates multiple metabolic enzymes, immune regulators, and additional transcription factors, including the key factor SKN‐1 (Wu et al., 2018). Importantly, ATF5 (the mammalian homolog of ATFS‐1) has been shown to regulate mammalian UPR^mt^ in a similar manner to ATFS‐1, indicating significant functional conservation between worms and humans (Fiorese et al., 2016; Qureshi et al., 2017). One interesting question prompted by these studies is how UPR^mt^ chaperones can be efficiently imported to resolve the stress conditions, when that failed process is what drives their production in the first place. Recent work by multiple laboratories has begun to address this question (Rolland et al., 2019; Shpilka et al., 2021; Xin et al., 2022) and indicates that the comparatively weak mitochondrial targeting sequence of ATFS‐1, at least compared to other proteins, causes its redirection.

The UPR^mt^ collaborates with the ISR pathway to reduce general protein translation rate and, consequently, the incoming load of mitochondrial proteins. The ISR (Harding et al., 1999; Harding et al., 2003) is an elaborate adaptive response that involves specialized kinases to promote the phosphorylation of eukaryotic translation initiation factor 2 (eIF2α). Phosphorylated eIF2α blocks the formation of the 43S pre‐initiation complex, inhibiting protein synthesis, but activating the transcription of certain effectors (such as ATF4) to promote cell survival (Harding et al., 2003). The ISR responds to many different stimuli, including ROS generation due to dysfunctional mitochondria which induces GCN‐2‐dependent eIF2α phosphorylation (Baker et al., 2012). The ISR functions in cooperation with the ATFS‐1‐mediated response to help restore protein folding. GCN‐2 activity is required for lifespan extension due to mild mitochondrial dysfunction (Baker et al., 2012).

Interestingly, GCN‐2 is not the only kinase that responds to mitochondrial perturbation in the context of the ISR. A novel pathway, called the OMA1‐DELE1‐HRI pathway, was recently found in mammalian cells to relay mitochondrial stress to the cytosol (Guo et al., 2020). This pathway must first be activated to activate ATF4 of the ISR to handle mitochondrial stress (Guo et al., 2020). OMA1 is a protease that cleaves DELE1, an inner mitochondrial membrane‐associated protein, that is released to the cytosol and in turn interact with HRI, a kinase that will phosphorylate eIF2α, leading to the translation of ATF4 (Guo et al., 2020). In contrast to the GCN‐2‐dependent ISR pathway, the OMA1‐DELE1‐HRI pathway has opposing effects on cell survival depending on the type of mitochondrial stress. A different ISR pathway, mediated by DELE1 and HRI (but independent of OMA1), was also found to be activated due to stalled protein import during iron starvation (Sekine et al., 2022). DELE1 stabilization on the outer mitochondrial membrane allows for interaction with the kinase HRI, activating the ISR. This illustrates the utility of monitoring iron sensing via mitochondrial transport.

Similarly, in *Saccharomyces cerevisiae*, disturbances in proteostasis activate a UPR^mt^‐related stress response pathway called the UPR^am^. The UPR^am^ detects the accumulation of precursor proteins in the cytosol (Wrobel et al., 2015). Activation reduces protein synthesis to reduce the workload of the protein import system in an effort to restore proteostatic homeostasis. Global changes in transcription profiles to decrease mitochondrial protein load, such as repression of mitochondrial oxidative phosphorylation machinery gene expression, are also achieved by inactivating the HAP complex (CCAAT box‐containing proteins) (Boos et al., 2019).

Unexpectedly, Tom70 was found to regulate both the transcription and import of mitochondrial proteins as well (Liu et al., 2022). Tom70 overexpression increases the abundance of mitochondrial proteins and mtDNA, suggesting that Tom70‐mediated mitochondrial protein import may regulate the biogenesis of mitochondrial proteins. This effect is relayed by multiple pathways. For example, knockout of the Forkhead family of transcription factor Fkh1/2 or the addition of the ROS scavenger N‐acetylcysteine partially reduce the effect of Tom70 overexpression (Liu et al., 2022).

The UPR^am^ also functions to increase proteasomal activity, and so are other import‐sensitive pathways in yeast, such as the mitochondrial compromised protein import response (mitoCPR) (Weidberg & Amon, 2018) and the novel mitochondrial protein translocation‐associated degradation (mitoTAD) pathway (Figure 2). Clogging the protein import system immediately activates Hsf1, possibly due to the depletion of the pool of free chaperones, and induces the transcription of Rpn4 (Boos et al., 2019), a regulator of the proteasome system of the UPR^am^. In turn, Rpn4 regulates the transcription of Pdr3 of the mitoCPR system (Weidberg & Amon, 2018). Accumulation of proteins in the TOM/TIM channel activates Pdr3, which initiates the transcription of mitoCPR target genes Cis1 and Msp1, among others. This role is specific to Pdr3, which additionally functions somewhat redundantly with Pdr1 in the multidrug response to various xenobiotic toxins (Moye‐Rowley, 2003). Cis1 interacts with Tom70 as a scaffold to recruit Msp1 and the proteasome. Msp1 is an AAA ATPase that removes the stuck proteins, allowing their proteasomal degradation (Basch et al., 2020; Weidberg & Amon, 2018). It is worth noting that disruptions to phospholipid biogenesis can also trigger mitochondrial import stress and activate this pathway (Sam et al., 2021). Meanwhile, the mitoTAD pathway directly monitors the TOM channel for clogging (Mårtensson et al., 2019). Upon detection of clogging, this pathway imports Ubx2 into the mitochondria, which recruits Cdc48 (an AAA ATPase) to remove precursor proteins clogged in the import channel, ensuring that mitochondrial protein import continues at full capacity.

More interestingly, the accumulation of protein aggregates in the mitochondria also activates an early branch of the UPR^mt^ in yeast that is mediated by the transcription factor Rox1 (Poveda‐Huertes et al., 2020). This pathway is activated very early in the response, with the apparent goal of maintaining mitochondrial membrane potential, protein import, and protein translation to promote cell survival. In contrast to ATFS‐1, Rox1 is normally a nuclear transcription factor. When precursor protein aggregation is recognized, Rox1 relocates to the mitochondrial matrix, where it regulates mtDNA expression. This is thought to circumvent the need for processing, increasing the speed of the response.

In contrary, when the damage is irreparable, the cells activate pathways design to recycle mitochondria and/or limit damage. One of these pathways is mitophagy (mitochondrial autophagy, a clearance pathway for damaged mitochondria) (Pickrell & Youle, 2015). The serine–threonine kinase PINK‐1, a well‐known regulator of mitophagy, is likewise sensitive to mitochondrial import disturbance. Much like ATFS‐1, PINK‐1 is constitutively expressed, trafficked to mitochondria, and rapidly degraded in both *C. elegans* and mammals. Unlike ATFS‐1, PINK‐1 stays at mitochondria when import is compromised, whether by disruptions of the mitochondrial membrane potential or blockage of the TOM/TIM complex. PINK‐1 accumulates on the outside of the mitochondrial membrane, dimerizes and cross‐phosphorylates, activating the protein and allowing it to phosphorylate its targets, such as the E3 ubiquitin ligase Parkin (Kane et al., 2014; Kazlauskaite et al., 2014). This triggers polyubiquitination of its substrates, allowing them to be recognized as targets for mitophagy (Bertolin et al., 2013; Mouton‐Liger et al., 2017; Narendra et al., 2008; Narendra et al., 2010; Pickrell & Youle, 2015). Interestingly, when alterations (e.g., mutated PINK1 or the loss of Tom7) were introduced that allowed PINK1 to be imported into mitochondria despite the loss of membrane potential, the kinase is cleaved by OMA1 (Sekine et al., 2019), the same protease that is involved in the ISR. Upon cleavage, PINK1 is degraded by the proteasome. OMA1 suppression, however, cancels PINK1 import into the mitochondria and activates mitophagy, and therefore is considered as a potential therapy to stimulate mitophagy for neurodegenerative diseases.

In yeast, a novel mitochondria‐dependent cell death program, called the mPOS (mitochondrial precursor over‐accumulation stress) (Wang & Chen, 2015), is also activated by defects in mitochondrial import; specifically, the accumulation of precursor proteins in the cytosol. This pathway can also be activated by increased heteroplasmy, protein misfolding, or reduced mitochondrial membrane potential (Coyne & Chen, 2018). Several genes were identified to suppress mPOS, including portions of the TOR pathway, mRNA turnover, reduced protein translation, and tRNA methylation (Wang & Chen, 2015). Like UPR^mt^ and UPR^am^, the suppressors of mPOS are targeted toward recovery of homeostasis, rather than directly activating cell death pathways.

The heavy reliance mitochondria have on protein import requires close observation and immediate response to possible dysfunction, especially as mitochondrial precursor proteins are prone to aggregation (Nowicka et al., 2021). In mammals and *C. elegans*, the UPR^mt^ plays a prominent role to ensure that mitochondrial proteostasis is restored. In yeast, multiple pathways have been identified within the last decade for resolving problems in protein import. The UPR^am^ and mitoCPR work harmoniously with the proteasomal system to remove problematic precursor proteins from the clogged import systems. Recently discovered pathways, such as the OMA1‐DELE1‐HRI, iron‐sensing DELE1‐HRI, early UPR^mt^, and mitoTAD pathways, represent the wide variety of surveillance targets in the mitochondrial protein import systems. It remains to be determined whether these pathways also activate mitophagy and programmed cell death pathways like their more well‐understood cousins, but it is an area of considerable interest.

## 3 DAMAGING THE ELECTRON TRANSPORT CHAIN: ROS AS A SIGNAL

ATP generation in mitochondria involving the ETC comes with a downside: The system is leaky, allowing electrons to escape from different carriers (e.g., NADH, FADH_2_, and coenzyme Q) and reduce O_2_ into superoxide (O_2_^•−^) (Quinlan et al., 2013), making mitochondria the largest single source (~90%) of ROS in the cell (Nissanka & Moraes, 2017). Once generated, ROS can damage most biomacromolecules, including proteins, lipids, and nucleic acids (Checa & Aran, 2020). Predictions of intracellular ROS were made as early as 1956 (Harman, 1956) and were supported by the discovery of superoxide dismutase (McCord & Fridovich, 1969), which converts superoxide into hydrogen peroxide.

Fascinatingly, increased mitochondrial superoxide due to mitochondrial ETC knockdown or mitochondrial superoxide dismutase deletion increases lifespan in *C. elegans* (Schaar et al., 2015; Van Raamsdonk & Hekimi, 2009). Similar effects were also observed in mice (Lapointe et al., 2012) and yeast (Pan et al., 2011). This indicates that ROS are not merely a toxic byproduct that needs to be eliminated. Instead, the production of mitochondrial ROS is critical for cell signaling and immune responses (Moldogazieva et al., 2018; Pinegin et al., 2018).

Mitochondrial ROS are known to activate the Nrf2 oxidative stress response pathway (Kasai et al., 2020) and the TOR pathway (Schieber & Chandel, 2014), a nutrient‐sensing pathway for cell growth and proliferation. Metabolic adaptations occurring due to the activation of these pathways are implicated in lifespan extension. The Nrf2 pathway may provide its beneficial effects by maintaining mitochondrial homeostasis, such as the expression of antioxidant and mitochondrial quality control genes. Similarly, TOR signaling senses mitochondrial ROS released by transient exposure to hypoxia, leading to the expression of detoxification genes, such as glutathione S‐transferases (Schieber & Chandel, 2014). Further, increased hydrogen peroxide production by mitochondria is known to stabilize the hypoxia‐inducible transcription factors (HIF) during hypoxia. This response is regulated by HIF‐1 and AMP‐activated protein kinase (AMPK) in a feedback regulation manner. HIF regulates transcription of genes encoding cell cycle regulators, innate immune effectors, and other key factors (Hamanaka & Chandel, 2010; Hwang et al., 2014).

The modulation of ROS levels is known to determine physiological outcomes. For example, low levels of ROS can activate the production of antioxidants to repair homeostasis, a process often known as mitochondrial hormesis (mitohormesis) (Hekimi et al., 2011; Ristow & Zarse, 2010). Elevated mitochondrial ROS production, known as a respiratory or oxidative burst, is also used as a cellular defense mechanism after pathogen engulfment or invasion. This response has both bactericidal (West et al., 2011) and long‐range signaling properties, for example, to promote wound repair (Xu & Chisholm, 2014), but high levels of ROS are detrimental to cellular survival.

## 4 MITOCHONDRIAL ROS‐RESPONSIVE PATHWAYS MONITOR REDOX STATUS

The biphasic effect of mitochondrial ROS suggests that cells possess surveillance systems that track cellular redox status to provide protection for the cell. Cells maintain pools of redox pairs (e.g., NADH/NAD^+^ or GSH/GSSG) to help mitigate ROS, but excessive ROS depletes the reductive member of these pairs, a condition called oxidative stress. Depletion of these pools causes accumulation of ROS and damage to mtDNA and proteins, accelerating mitochondrial dysfunction. Mitochondrial ROS may promote calcium release from the endoplasmic reticulum (ER) and trigger additional ROS production from surrounding mitochondria (Bertero & Maack, 2018). Ultimately, the overload of oxidative agents can trigger the opening of the mitochondrial permeability transition pore, energetic collapse, cytochrome *c* release, and cell death (Jacobson & Duchen, 2002).

Although oxidative stress has been a focus of many studies, it is not the only consequence of mitochondrial disruption. An abnormal buildup of reducing equivalents, especially NADH, NADPH, or GSH, leads to a state called reductive stress. This can occur when Complex I of the ETC is disrupted, preventing NADH oxidation. Paradoxically, reductive stress also leads to production of ROS, as molecular oxygen is reduced (yielding superoxide) when more typical electron acceptors are absent (Korge et al., 2015; Zhang et al., 2012). Production of ROS from either oxidative or reductive stresses is dangerous to cells (Brewer et al., 2013; Xiao & Loscalzo, 2020). Moderate induction of reductive stress, however, drives mitochondrial hormesis to prepare for defense against oxidative stress (Singh et al., 2015; Spanidis et al., 2018).

**FIGURE 3:**
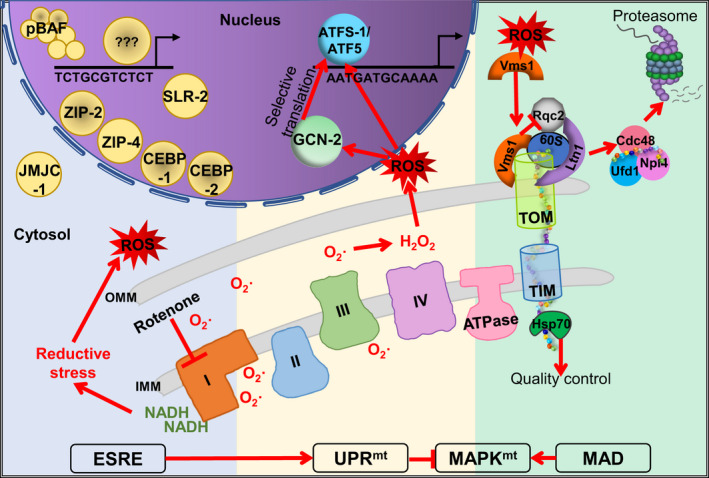
Mitochondrial ROS surveillance pathways. Three ROS‐responsive pathways are illustrated, from left to right. First, the ESRE pathway, which is triggered by superoxide, involves multiple transcription factors, the PBAF chromatin remodeling complex, and JMJC‐1 to regulate the expression of ESRE genes (i.e., genes with the 11‐nucleotide TCTGCGTCTCT motif in their promoter region). Abnormal buildup of reducing equivalents (due to mitochondrial disruption) that paradoxically increases ROS production can also activate the ESRE pathway. Second, ROS accumulation induces GCN‐2‐dependent eIF2ɑ phosphorylation, altering translational profile, and working in concert with the UPR^mt^ transcription factor ATFS‐1/ATF5 to restore proteostasis. Third, oxidative stress triggers Vms1 of the MAD pathway, to translocate from the cytosol to the OMM. Once there, it recruits the ribosomal quality control complex to help release proteins whose translocation has stalled for proteasomal degradation. Abbreviations: IMM—Inner mitochondrial membrane, OMM—outer mitochondrial membrane, ROS—reactive oxygen species, TOM/TIM—translocase of the outer/inner membrane

Mitochondrial surveillance pathways that are responsive to ROS and/or redox stress have received increasing attention over the last decade (Figure 3). One of the first ROS‐responsive pathways discovered was the mitochondrial‐associated protein degradation (MAD) pathway, first identified in yeast (Heo et al., 2010). Upon detection of oxidative stress, Vms1, which is highly conserved among eukaryotes, translocates from the cytosol to the outer mitochondrial membrane. Once there, it recruits and interacts with the ribosomal quality control complex (comprised of Rqc1, Rqc2, Ltn1, Cdc48, Ufd1, and Npl4) (Verma et al., 2018). Vms1 also binds the 60S ribosome and facilitates release of stalled translation of mitochondrial proteins (Izawa et al., 2017). The cytosolic 26S proteasome was speculated to be redirected to the mitochondria to help with the degradation of these proteins (Segref et al., 2014). The importance of this pathway is shown in Vms1 loss‐of‐function mutants, which display reduced cellular viability and mitochondrial function and increased sensitivity to oxidative stress (Heo et al., 2010).

*C. elegans* UPR^mt^ has also been reported to respond to ETC disruptions that cause ROS (Runkel et al., 2013). Interestingly, this response is different from the regular UPR^mt^, as it involves neither the transporter HAF‐1 nor peptide efflux. Induction of this response can be suppressed by the mutation of over 50 genes, including regulatory subunits of the proteasome, ribosomal components, chaperones, and the transcription factors ATFS‐1 and ELT‐2. Most of these genes are also associated with the cellular surveillance activated detoxification and defenses (cSADD) program that monitors disruption of basic cellular functions (Melo & Ruvkun, 2012; Runkel et al., 2013). The authors speculated that the repression by cSADD may indicate that cells can temporarily repress the UPR^mt^ in order to focus on resolving more immediate threats or to temporarily increase local ROS levels for an “active burst” immune response as part of a defense strategy (Runkel et al., 2013).

Another key cellular response activated by ROS is the ESRE network. Initially identified by its activation after acute ethanol exposure, the ESRE pathway was named for an 11‐nucleotide motif (TCTGCGTCTCT), known as the ethanol and stress response element (ESRE), that is present in the promoter region of responsive genes (Kwon et al., 2004). Interestingly, the ESRE motif has since then been independently discovered at least seven times in studies of stress responses in *C. elegans* and in mammals, and has been shown to be activated in response to hypoxia, ethanol, heat, and oxidative stress (Gaudet et al., 2004; GuhaThakurta et al., 2002; Kirienko & Fay, 2010; Kwon et al., 2004; Munkácsy et al., 2016; Pignataro et al., 2007; Ruvinsky et al., 2007). In many of these instances, the removal or mutation of ESRE motif(s) from the promoter of responsive genes abolishes their expression (Gaudet et al., 2004; GuhaThakurta et al., 2002; Kwon et al., 2004; Pignataro et al., 2007; Tjahjono & Kirienko, 2017). Later work by our group showed that the ESRE network also responds to mitochondrial damage inflicted by the removal of iron by either a bacterial siderophore or by a chemical iron chelator (Kang et al., 2018; Tjahjono & Kirienko, 2017). We anticipate that activation of the ESRE network in each of these cases results from mitochondrial damage triggering the production of superoxide anions. It is worth nothing that exposure to a broad variety of poisons that damage the ETC activates the ESRE response. One example is rotenone, which prevents electron transfer from NADH and causes it to accumulate, inducing reductive stress. As might be expected, adding *N*‐acetylcysteine (a well‐known antioxidant) increases the reductive stress and amplifies the ESRE response (Tjahjono et al., 2020).

The ESRE network is not restricted to nematodes, but is broadly evolutionarily conserved (Kirienko & Fay, 2010). Importantly, genes regulated by the ESRE motif in *C. elegans* typically retain the motif across large evolutionary distances (i.e., between *C. elegans* and humans), are often orthologous between humans and nematodes, and frequently are involved in stress responses (Kirienko & Fay, 2010). The ESRE motifs are also found in the promoter of *atfs‐1* and *bec‐1*/Beclin, regulators of UPR^mt^ and autophagy pathways in *C. elegans*, respectively. As was seen for other genes, deletion of the ESRE motif from the promoter of *atfs‐1* reduced the expression of this gene (Tjahjono et al., 2020), affirming ESRE's role in the regulation of important pro‐mitochondrial health pathways. As such, considerable attention has been given to understanding how superoxide is detected and how this drives transcriptional activity. The most obvious explanation is that one (or more) transcription factor(s) bind to the ESRE site, which is upstream of predicted transcriptional start sites. However, attempts to identify candidate proteins (via targeted RNAi screens or biochemical purification of transcription factors) have thus far been unsuccessful ([Kuzmanov et al., 2014], N. V. Kirienko, personal communication). Despite this, we have shown that at least four C/EBP bZip family transcription factors (ZIP‐2, ZIP‐4/CEBPβ, CEBP‐1, and CEBP‐2/CEBPγ) play roles in ESRE gene regulation (Tjahjono & Kirienko, 2017). The nematode‐specific Zn‐finger transcription factor SLR‐2 also regulates ESRE gene expression (Kirienko & Fay, 2010), but ESRE gene activation was seen in strains carrying mutations predicted to have strong loss‐of‐function alleles in all of these transcription factors, suggesting that ESRE expression only partially depends on any of these genes. To date, no single transcription factor has been shown to be indispensable for ESRE activity.

ESRE gene expression also requires the PBAF chromatin remodeling complex (Kuzmanov et al., 2014), which recognizes highly acetylated chromatin (Ho et al., 2019). Elements of the PBAF complex (SWSN‐4/BRG1/BRM and SWSN‐1/BAF170/BAF155) appear to bind to the promoters of ESRE‐containing genes, even in the absence of stress (Riedel et al., 2013), while other elements (SWSN‐7 and PBRM‐1) are stress‐inducible (Kuzmanov et al., 2014). Overexpression of the stress‐inducible portions of the PBAF complex increased expression of ESRE genes, even in the absence of stress, and increased stress resistance. Interestingly, the PBAF complex was only recruited to intact ESRE sites; removal of the ESRE motif abolished binding by the nucleosome remodeling complex, indicating that the site itself is necessary for recruitment.

Another recent study identified box C/D snoRNA (small nucleolar RNA) core proteins (snoRNPs) as ESRE interactors (Tjahjono et al., 2022). Box C/D snoRNPs are comprised of FIB‐1/Fibrillarin (the catalytic methyltransferase), NOL‐56/Nop56, NOL‐58/Nop58, and M28.5/SNU13. Box C/D snoRNPs 2’‐O‐methylate RNAs, especially rRNA, in a sequence‐dependent fashion, using snoRNAs for targeting and sequence recognition (Ojha et al., 2020). Multiple members of this protein complex were identified as directly binding ESRE element in an oligo pull‐down experiment. Based on follow‐up experiments, authors proposed a model where box C/D snoRNP machinery may function as a “switch” of the cell's activity between mitochondrial surveillance and innate immune activation, as mutations in these genes resulted in decreased mitochondrial function and upregulation of innate immune pathways (Tjahjono et al., 2022).

Another factor involved in ESRE gene expression is an enzyme called JMJC‐1/RIOX1/NO66 (Kirienko & Fay, 2010). JMJC‐1/RIOX1/NO66 is a member of the Jumonji family of proteins, which contains over 30 members, most of which have demonstrated histone demethylase activity (Franci et al., 2014). The molecular function of RIOX1 is less clear, but has been very capably reviewed (Bundred et al., 2018). It has been convincingly demonstrated to transfer a hydroxyl group to a histidine in the ribosomal protein Rpl8 (Ge et al., 2012; Williams et al., 2014) and there is some evidence that it may have histone demethylase activity (Bundred et al., 2018; Sinha et al., 2010; Zhou et al., 2012), although this activity is controversial as it could not be recapitulated by other groups (Wang et al., 2015; Williams et al., 2014). While demethylation has an obvious mechanism for regulating gene expression (i.e., the conversion of chromatin to a more readable state), ribosomal modification is less clear. One careful structural study indicates that the transfer helps stabilize the local conformation of the 28S rRNA and the peptidyl transfer center, and has been proposed to enable translational efficacy (Yanshina et al., 2015). Parsing out these functions in vivo is difficult as both functions utilize the same chemistry, coordinated by the same amino acid residues. This remains an active area of study.

Disrupting the mitochondrial ETC has recently been shown to activate several other responses as well. For example, RNAi knockdown of *cox‐6c*, a component of Complex IV, caused dephosphorylation of HSF‐1 by LET‐92 (Williams et al., 2020). Interestingly, overexpression of *let‐92* supported proteostatic health and limited aggregation‐induced paralysis in worms carrying a glutamate‐repeat protein. Dephosphorylated HSF‐1, at least in these conditions, primarily drove the expression of small, ATP‐independent heat shock proteins that are thought to sequester misfolded proteins while waiting for an ATP‐dependent chaperone to refold them. HSF‐1 dephosphorylated in this fashion also upregulates two HSP70 family members, HSP‐70 and HSP‐70B, that have this function, even though their function is likely to be limited while the ETC is disrupted. The authors hypothesized that this upregulation poises the system to recover quickly once ATP has begun to be produced (Williams et al., 2020). A subsequent study showed that exposing *C. elegans* to a variety of compounds (including acivicin, cadmium, or acetaminophen) disrupted the balance of cellular redox compounds by depleting the pool of thiols (Gusarov et al., 2021). This study also demonstrated that excess consumption of antioxidants, such as *N*‐acetylcysteine, can similarly disrupt cellular redox balance and, at least in *C. elegans*, shorten lifespan.

Another important mechanism of maintaining ETC function is turnover of its damaged components by matrix‐resident proteases, especially the AAA protease SPG‐7/SPG7 (Arlt et al., 1996). RNAi‐mediated disruption of *spg‐7* has been clearly shown to activate several mitochondrial surveillance pathways (Munkácsy et al., 2016; Yoneda, 2004), including the UPR^mt^ and, if *atfs‐1* is compromised, the ESRE network as a compensatory mechanism (Tjahjono et al., 2020). Interestingly, *atfs‐1* mutants also showed upregulation of a second pathway, which was dependent upon a DLK‐1/SEK‐3/PMK‐3 MAPK pathway (Munkácsy et al., 2016). Using a transcriptional reporter for *tbb‐6*, one of the most highly upregulated genes after disruption of *spg‐7*, they showed that the MAPK^mt^ system is activated by a variety of mitochondrial bioenergetic perturbations. Disruption of the MAD pathway reduced expression of the *Ptbb‐6*::GFP reporter, indicating that the MAPK^mt^ system may be activated downstream of the MAD pathway, possibly through cytosolic signaling that was stabilized upon reduction of the ubiquitin/proteosome system activity. Much like the ESRE network, expression of MAPK^mt^ target genes appears to at least partially depend on C/EBP family transcription factors; removal of C/EBP‐like binding motifs in the *tbb‐6* promoter abolished induction of the reporter (Munkácsy et al., 2016).

As the core component of the mitochondria, it is far from surprising that multiple pathways are dedicated to surveilling the integrity of the ETC. The MAD, the UPR^mt^, and the ESRE pathways directly respond to shifts in redox balance, while the others may monitor other damaged sites. Careful modulation of ROS production and redox conditions is crucial to potentiate ROS as signaling molecules. As ROS have myriad functions in cell signaling, discovering novel signaling pathways dependent on ROS and redox conditions may help to leverage mitohormesis to improve organismal fitness.

## 5 LIPID DYSREGULATION: THE NEXT FRONTIER IN MITOCHONDRIAL SURVEILLANCE?

Mitochondrial functions are tightly linked with lipid metabolism and signaling. For example, most β‐oxidation of fatty acids takes place in the matrix. Other mitochondrial metabolic activities also generate signaling lipids that play roles in mitophagy, autophagy, and apoptosis (Crimi & Esposti, 2011; Dall'Armi et al., 2013; Nielson & Rutter, 2018). Unsurprisingly, a genome‐wide screen identified RNAi of lipid biosynthesis genes to trigger the activation of the mitochondrial chaperone HSP‐6 (Liu et al., 2014). This screen also identified a wide variety of genes, including known components of the UPR^mt^, nuclear pore and transport machinery, and kinases and phosphatases. Most of these knockdowns also activated reporters for xenobiotic detoxification and pathogen response, which led them to conclude that *C. elegans* interprets mitochondrial dysfunction as a xenobiotic exposure or a pathogen attack. This is consistent with their findings that a wide range of bacteria encountered by *C. elegans* in their natural environment damage host mitochondria.

Their data also indicated that mitochondrial surveillance in these circumstances required SPTL‐1, a key protein in sphingolipid biosynthesis, and the mevalonate biosynthesis protein HMGS‐1. Moreover, they showed that supplementation with a 24‐carbon ceramide, a downstream product of SPTL‐1, rescued mitochondrial surveillance. Previous reports indicated that loss‐of‐function of mutation of HYL‐2, the protein that synthesizes 24‐ and 26‐carbon ceramides, triggered autophagy in *C. elegans* (Mosbech et al., 2013), possibly due to failures in mitochondrial surveillance.

**FIGURE 4:**
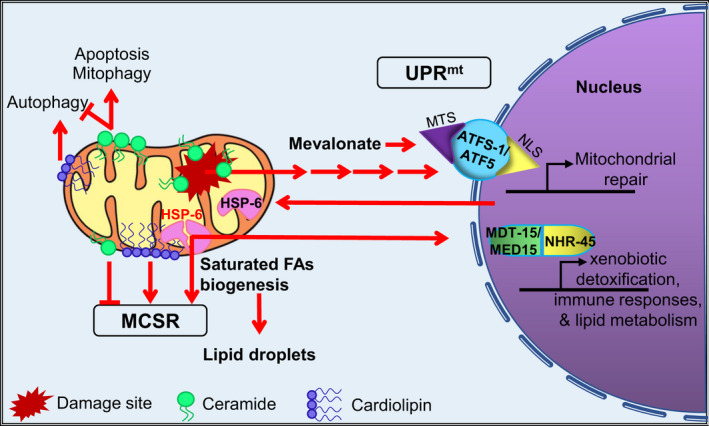
Lipids surveillance in mitochondria. At least two pathways, the UPR^mt^ and the MCSR, involve monitoring the levels of various lipid species. For example, sites of mitochondrial damage are marked by ceramides, which activate the UPR^mt^. Mevalonate, a precursor of many biologically important molecules, such as cholesterol, ubiquinones, and heme a, is also implicated in UPR^mt^ regulation. Accumulation of ceramides on the outer mitochondrial membrane promotes apoptosis and mitophagy while inhibiting autophagy. Ceramides also block the MCSR, a cytosolic stress response that is triggered by mitochondrial dysfunction. In contrast, cardiolipin (a lipid largely restricted to mitochondria in eukaryotes) activates the MCSR and promotes autophagy. Disruption of HSP‐6 also triggers the formation of lipid droplets, a stress response to minimize lipotoxicity, and activates a xenobiotic response mediated by MDT‐15/NHR‐45. Abbreviations: FA—fatty acid, MTS—mitochondrial targeting sequence, and NLS—nuclear localization signal

It should be noted that HSP‐6, the mitochondrial chaperone used in that study, occupies a rather unique space in *C. elegans*. For example, a hypomorphic allele of *hsp‐6* activates a xenobiotic response through MED‐15 and NHR‐45, two proteins associated with lipid metabolism (Mao et al., 2019). Additionally, Kim et al. observed that disruption of *hsp‐6*, which activates the UPR^mt^, also activates the heat shock response in the cytoplasm, a phenomenon they named the mitochondrial‐to‐cytosolic stress response, or MCSR (Kim et al., 2016; Figure 4). In contrast, RNAi targeting other known mitochondrial chaperones (e.g., *hsp‐60 and dnj‐10*) did not have a similar effect. The reason for this difference remains unknown.

Induction of the MCSR after *hsp‐6(RNAi)* was blocked if either *pod‐2* or *fasn‐1*, two genes early in the biosynthetic pathway for saturated fatty acids, were knocked down with *hsp‐6*. Lipid profiling demonstrated that the MCSR was associated with decreased ceramide biosynthesis and increased cardiolipin, two lipid groups whose concentrations are often inversely correlated. Interestingly, they observed that merely feeding exogenous cardiolipin to worms was sufficient to trigger mild activation of the heat shock response and activate HSP‐6, and that *cris‐1(RNAi)*, which knocks down the cardiolipin synthase gene, blocked MCSR. Both observations further link lipid biology to this stress response. This effect may have been indirect, however, as their work indicated that the absence of ceramide may actually be more important to the activation of the MCSR than the presence of cardiolipin (Kim et al., 2016).

While studying the MCSR, they observed that *hsp‐6(RNAi)* also triggered the accumulation of lipid droplets (Papsdorf & Brunet, 2019). The formation of these bodies has itself been described as a stress response, a mechanism to minimize lipotoxicity. For example, increased autophagic activity in mammalian cells, especially during starvation, upregulates lipid metabolism. This results in the production of large pools of acylcarnitines, a class of lipids that are responsible for the transport of fatty acids into mitochondria for β‐oxidation. However, high concentrations of acylcarnitines have been linked with lipotoxicity and mitochondrial dysfunction (McCoin et al., 2015; Son et al., 2010; Wajner & Amaral, 2015). This effect can be limited by the action of the lipid metabolism gene, DGAT1, which converts acylcarnitines into more easily‐stored triglycerides, which are then packed into lipid droplets (Nguyen et al., 2017). DGAT1 is upregulated during autophagy, and its absence leads to considerable mortality during starvation when autophagy is activated (Nguyen et al., 2017).

Although these data clearly indicate a relationship between lipid metabolism and mitochondrial surveillance, it should be noted that these data present two possibilities. First, it is possible that altered lipid metabolism directly disrupts homeostasis for mitochondria, the ER, or some other organelles. For example, fatty acids were shown to alter mitochondrial membranes permeability and inhibit ETC complexes (Penzo et al., 2004; Schönfeld & Wojtczak, 2008). Inappropriate lipid metabolism may also physically disrupt organelles by acting as membrane detergents, or the failed production of lipid droplets may prevent the removal of inappropriate fatty acid species from the ER or other organelles (Roberts & Olzmann, 2020). A second alternative is that one or more lipids serves as a signal for mitochondrial health. This signal could take the form of either an “all clear” signal that stops being produced during stress conditions or a danger signal that is produced, or accumulates, during mitochondrial disruption. For example, mitochondrial damage has been shown to trigger the relocalization of cardiolipin from the inner mitochondrial membrane to the outer membrane, allowing the phospholipid to facilitate recognition of mitochondria as an autophagosomal target (Chu et al., 2013). In any case, it is increasingly clear that the roles of lipids in mitochondrial surveillance demand further attention.

## 6 SURVEILLANCE PROGRAMS PROMOTE HEALTHY MITOCHONDRIA AND LIFESPAN EXTENSION

Artificial perturbation of the mitochondrial environment, for example, by knocking down resident proteins or chemically inhibiting ETC complexes, led to the discovery of many of the mitochondrial surveillance mechanisms described above. While studies under these conditions have provided mechanistic insight, a discussion of how these pathways function in more natural contexts, such as aging and immunity, is warranted. Artificial perturbations that activate mitochondrial surveillance generally recapitulate the environment in aging cells. For example, accumulation of mitochondrial ROS and downstream oxidative stress‐modified molecules are common biomarkers of aging and aging‐related diseases (Frijhoff et al., 2015). Furthermore, declines in mitochondrial quality have been increasingly recognized to contribute to aging and the development of aging‐associated and other chronic diseases, including cardiovascular diseases, diabetes, and obesity. MtDNA deletions and rearrangements are increased among elderly individuals and are also primary cause of the Kearns–Sayre syndrome, POLG‐related disorders, and multiple sclerosis in which symptoms resemble premature aging (Corral‐Debrinski et al., 1992; Poulton et al., 1994; Rygiel et al., 2016). In aging heart, oxidative phosphorylation and beta‐oxidation are reduced, leading to reduced ATP production but increased lipid and ROS (Lesnefsky et al., 2016). These abnormalities result in inflammation and degenerated functions of affected tissues, which are the primary hallmarks of aging. As such, it is important to understand the substantial roles of the mitochondrial surveillance pathways in aging and immunity.

The study of mitochondrial surveillance resulted in a fine observation that careful modulation of mitochondrial perturbation could be beneficial to the cell. Research in *C. elegans* repeatedly showed that moderate mitochondrial ETC inhibition extends lifespan (Dillin et al., 2002; Lee et al., 2003; Rea et al., 2007). Prolonged perturbation, however, causes cell damage or death and the release of mtDAMPs that rapidly triggers immune responses. Thus, mitochondrial dysfunction is one of the hormetic phenomena in aging (López‐Otín et al., 2013). We have now understood that this antagonistic characteristic of mitochondrial perturbation outcomes is determined by mitochondrial quality control mechanisms. Mitochondrial surveillance pathways constantly monitor mitochondria status indicators to prepare for appropriate response upon detection of abnormality. For example, the inhibition of mitochondrial ETC increases ROS production, leading to the induction of various mitochondrial surveillance pathways that further activate detoxification systems and stress responses. The induction of surveillance pathways enables early detection of damage and makes appropriate decision for homeostasis restoration effort. When these efforts seem to be futile, mitochondria may undergo self‐degradation to limit the propagation of sick mitochondria and to recycle their components.

The importance of mitochondrial surveillance pathways in longevity and aging‐related diseases is evident as the loss of these pathways often results in repressed lifespan extension phenotype and/or reduced survival during stress. For example, ATFS‐1 of the UPR^mt^ and PMK‐3 of the MAPK^mt^ pathway are required for the long lifespan observed in *C. elegans* Mit mutants (Munkácsy et al., 2016; Wu et al., 2018). The UPR^mt^ has also recently been linked to mitochondrial recovery upon starvation (Naresh et al., 2022) and its activation restored mitochondrial protein homeostasis in multiple Parkinson's disease models (Hu et al., 2021). The induction of MCSR and mitophagy improve proteostasis as shown in the accumulation of fewer aggregates in a Huntington's disease model in both *C. elegans* and mammalian cells (Kim et al., 2016; Tjahjono et al., 2021). The PINK‐1/Parkin mitophagy pathway has long been implicated in neurodegenerative diseases, especially Parkinson's disease (extensive review in [Mouton‐Liger et al., 2017]).

These surveillance pathways have equivalently profound roles in innate immune activation. The UPR^mt^ plays a protective role in response to pathogen exposure as expression of innate immune genes (e.g., lysozymes and anti‐microbial peptides) is orchestrated by ATFS‐1 (Pellegrino et al., 2014). Similarly, knockdown of genes belonging to the ESRE network reduce survival in a pyoverdine‐dependent *Pseudomonas aeruginosa* pathogenesis assay (Tjahjono & Kirienko, 2017). Finally, activation of the p38 MAPK immune pathway due to rotenone exposure confers neuroprotection through the activation of mitophagy, establishing a relationship between the two (Chikka et al., 2016).

It is also important to note that the surveillance programs do not act individually. Repression of the MAPK^mt^ by the UPR^mt^ and repression of the UPR^mt^ by cSADDs, presence of ESRE motif in the *atfs‐1* promoter, for example, suggest extensive crosstalk and illustrate the complexity of stress and surveillance regulations. These interactions extend beyond mitochondria; for instance, the canonical p38 MAPK immune signaling pathway is also involved in the increased resistance of the Mit mutants to pathogens (Campos et al., 2021). As with all surveillance pathways, the protective effects and lifespan extension occurred with mild induction of these retrograde response systems, while the opposite occurred with chronic pathway activation (Hsu et al., 2003; Labunskyy et al., 2014; Rea et al., 2007).

Finally, many questions remain on the roles of mitochondrial surveillance in promoting healthy aging and immunity. For example, how do mitochondrial surveillance pathways ameliorate proteostatic defects in the context of degenerative diseases? How does impaired mitochondrial surveillance lead to oncogenesis? Therefore, future research regarding the roles of signaling molecules, pathway modulations, and crosstalk in surveillance systems is necessary. This is not only crucial for understanding cell biology and aging regulations but may also have a huge potential for the development of novel therapeutic systems for healthy aging.

## AUTHOR CONTRIBUTIONS

ET involved in investigation, writing, review and editing, and visualization. DRK involved in writing, review and editing, and visualization. NVK involved in writing, review and editing, supervision, and funding acquisition.

## CONFLICT OF INTEREST

The authors have declared that no competing interests exist.

## Data Availability

N/A
